# Two Receptor Binding Strategy of SARS-CoV-2 Is Mediated by Both the N-Terminal and Receptor-Binding Spike Domain

**DOI:** 10.1021/acs.jpcb.3c06258

**Published:** 2024-01-08

**Authors:** Michele Monti, Edoardo Milanetti, Myrthe T. Frans, Mattia Miotto, Lorenzo Di Rienzo, Maksim V. Baranov, Giorgio Gosti, Arun Kumar Somavarapu, Madhu Nagaraj, Thaddeus W. Golbek, Emiel Rossing, Sam J. Moons, Thomas J. Boltje, Geert van den Bogaart, Tobias Weidner, Daniel E. Otzen, Gian Gaetano Tartaglia, Giancarlo Ruocco, Steven J. Roeters

**Affiliations:** † RNA Systems Biology, Centre for Human Technologies (CHT), Istituto Italiano di Tecnologia (IIT), Via Enrico Melen, 83, 16152 Genova, Italy; ‡ Center for Life Nanoscience, Istituto Italiano di Tecnologia, Viale Regina Elena 291, 00161 Rome, Italy; § Department of Physics, Sapienza University, Piazzale Aldo Moro 5, 00185 Rome, Italy; ∥ Molecular Immunology—Groningen Biomolecular Sciences and Biotechnology, Nijenborgh 7, 9747 AG Groningen, The Netherlands; ⊥ DHILab, Istituto di Scienze del Patrimonio Culturale, Sede di Roma, Consiglio Nazionale delle Ricerche, Via Salaria km, 29300, 00010 Rome, Italy; # Interdisciplinary Nanoscience Center (iNANO), Aarhus University, Gustav Wieds Vej 14, 8000 Aarhus C, Denmark; ¶ Department of Chemistry, Aarhus University, Langelandsgade 140, 8000 Aarhus C, Denmark; ∇ Synthetic Organic Chemistry, Radboud University Nijmegen, Heyendaalseweg 135, 6525 AJ Nijmegen, The Netherlands; ○ Amsterdam UMC, Vrije Universiteit, Department of Anatomy and Neurosciences, De Boelelaan 1108, 1081 HZ Amsterdam, The Netherlands

## Abstract

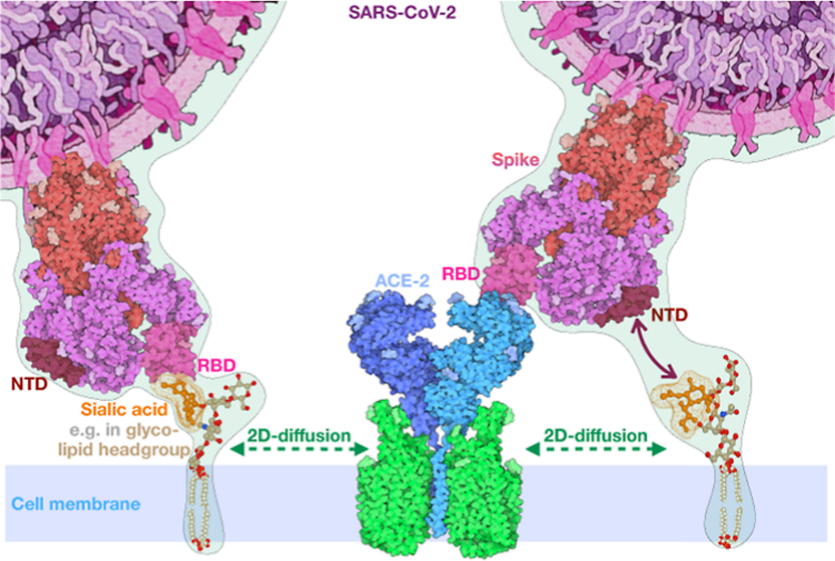

It is not well understood why severe acute respiratory syndrome (SARS)-CoV-2 spreads much faster than other β-coronaviruses such as SARS-CoV and Middle East respiratory syndrome (MERS)-CoV. In a previous publication, we predicted the binding of the N-terminal domain (NTD) of SARS-CoV-2 spike to sialic acids (SAs). Here, we experimentally validate this interaction and present simulations that reveal a second possible interaction between SAs and the spike protein via a binding site located in the receptor-binding domain (RBD). The predictions from molecular-dynamics simulations and the previously-published 2D-Zernike binding-site recognition approach were validated through flow-induced dispersion analysis (FIDA)—which reveals the capability of the SARS-CoV-2 spike to bind to SA-containing (glyco)lipid vesicles, and flow-cytometry measurements—which show that spike binding is strongly decreased upon inhibition of SA expression on the membranes of angiotensin converting enzyme-2 (ACE2)-expressing HEK cells. Our analyses reveal that the SA binding of the NTD and RBD strongly enhances the infection-inducing ACE2 binding. Altogether, our work provides *in silico*, *in vitro*, and cellular evidence that the SARS-CoV-2 virus utilizes a two-receptor (SA and ACE2) strategy. This allows the SARS-CoV-2 spike to use SA moieties on the cell membrane as a binding anchor, which increases the residence time of the virus on the cell surface and aids in the binding of the main receptor, ACE2, via 2D diffusion.

## Introduction

In the past few years, the exceptionally fast transmission of the severe acute respiratory syndrome (SARS)-CoV-2 virus has led to millions of deaths all over the world.^1^ Although the symptoms of COVID-19 are less severe as compared to some other β-coronaviruses, its fast viral diffusion still causes the virus to clog public health systems across the world, in spite of effective vaccines. Seven coronavirus strains have been shown to infect humans.^2,3^ In the past 20 years, along with SARS-CoV-2, two other β-coronavirus have caused three of the most severe epidemics reported in the world: SARS-CoV, which causes the SARS,^4,5^ and MERS-CoV,^6^ which causes the Middle East respiratory syndrome (MERS). These three viruses share a close evolutionary history,^7^ but the infection and diffusion rates are different, which indicates that the SARS-CoV-2 virus has developed a different infection mechanism and/or pathology with respect to the other species. The initial binding partners that SARS-CoV and MERS interact with for the infection of cells are different. For SARS-CoV, the receptor is the angiotensin converting enzyme-2 (ACE2). Instead, for MERS-CoV, the infection is mediated by an initial binding to nine-carbon acidic monosaccharides typically found at the terminal position of glycan chains present in the cell membrane, such as sialic acid (SA), which serve as attachment factors before binding with its main entry receptor, dipeptidyl peptidase 4 (DPP4).^2,8^ These different receptors partially explain the distinct spreading efficiency of the two viruses.^9^ Herein, we would like to stress that we use the term “receptor” with regards to SA solely to indicate that it facilitates the initial binding and that it thereby relays the signal to be incorporated into the biochemical pathway that follows from subsequent ACE2 binding, following the definition of “receptor” used in refs (10)–^15^. The interaction between these viruses and human-cell receptors is being extensively studied to understand the diffusion mechanism and explain the differences in mortality/spreading rate.^16−18^ Even though the molecular and genomic differences between SARS-CoV-2 and the other β-coronaviruses are small, they have inflicted catastrophic effects to our societies to very different degrees.

**Figure 1:**
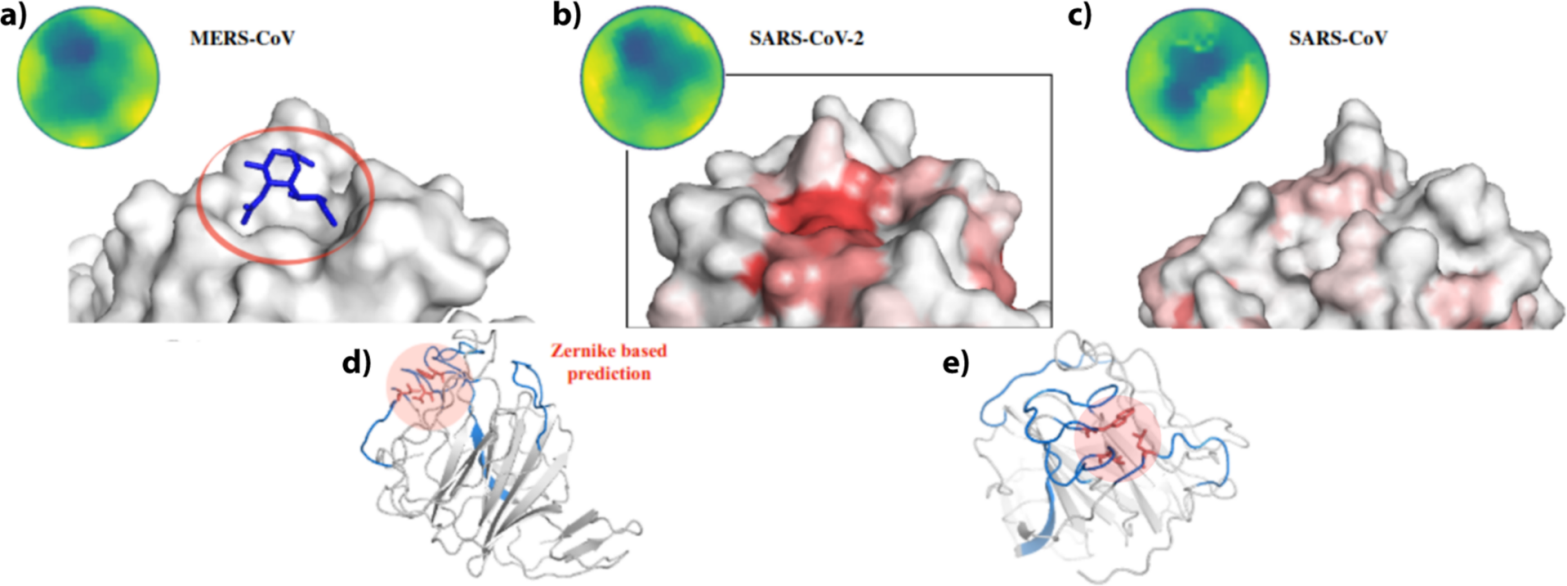
Characterization of a SARS-CoV-2 spike region very similar to the SA binding site on the MERS-CoV spike. (a) Molecular surface representation of the N-terminal region of the MERS-CoV spike bound to SA, along with the corresponding representative 2D-patch disk. (b) Molecular surface representation of the N-terminal region of SARS-CoV-2, colored from white to red according to their shape similarity with the MERS-CoV binding region, along with the corresponding representative 2D-patch disk. (c) Same as in panel b, but for SARS-CoV. (d) Cartoon representation of the SARS-CoV-2 NTD with the predicted binding region highlighted in red. (e) As in d, but rotated.

In our previous work,^19^ we identified how the spike of SARS-CoV-2 has evolved an N-terminal domain (NTD) SA binding pocket that is similar to the MERS-CoV one in the same genomic region (see Figure 1), which has recently been experimentally shown to interact with SA receptors.^12,20^

SAs are a class of α-keto acid sugars with a nine-carbon backbone. This small molecular moiety is omnipresent on all cell membranes of vertebrates and some invertebrates, mostly attached to the outermost ends of lipids and proteins that constitute the surface of the cell.^21^ These glycans are present in cells forming the external respiratory airways,^13^ so viruses like influenza-A^22,23^ use it as a receptor, while others like MERS-CoV^12^ exploit it as an initial, less specific, but ubiquitous attachment factor to enhance their infection rates through reducion of the 3D search for the main receptor to a 2D plane. The evolutionary advantage of this reduction in dimensionality relates directly to the findings obtained in our previous computational analysis based on the recently developed “2D-Zernike polynomial” formalism, which allows the characterization of the shape of portions of the molecular surface of proteins. With this method, we investigated the structural area around the SA binding site of MERS-CoV, confirming that SARS-CoV-2 has evolved a similar SA binding pocket in the NTD. This computational analysis shows the high similarity between the SA binding patch of the MERS-CoV spike, which was selected from the X-ray structure of the complex, and the corresponding patch of the SARS-CoV-2 spike located in the same NTD region. In contrast, the same analysis performed on the SARS-CoV spike shows its inability to bind SA, since no patches with similar geometric properties were found in comparison to the MERS binding site (see Figure 1). Importantly, the site interacting with SA is hypervariable and is mainly composed of disordered regions. The binding site belonging to the MERS spike has much more extensive disordered regions than the corresponding SARS-CoV region. Interestingly, the SARS-CoV-2 spike region has loops with an intermediate length between the MERS spike and the SARS-CoV spike. This suggests that the length of the SARS-CoV-2 loops is sufficient to bind with SA. Yet, the small differences between the MERS and SARS-CoV-2 regions could lead to a distinct binding affinity and thus to a different role of the SA receptor.

Previous studies have revealed that the receptor-binding domain (RBD) of the SARS-CoV-2 spike can also bind to SA.^23^ Here, we characterize and compare—both computationally and experimentally—the nature of the binding and the relative affinities between SA and the RBD and NTD as—until now—this has not been analyzed in detail. Using various techniques, we were able to reveal the mechanism of the binding and demonstrate its biological relevance.

From a computational perspective, to quantify the binding dynamics, we performed extensive molecular dynamics (MD) simulations of the system to quantify the binding dynamics. This computational analysis aims to reveal and compare the nature of the binding among the different domains at the atomic level. We ran the simulations on various spike segments, investigating the interaction of SA with the whole S1 chain of the spike and with the NTD and RBD.

From an experimental perspective, we performed experiments at different scales. We used two different techniques to characterize the strength and the biological effect of the binding between SA and spike proteins. On the one hand, we have used flow-induced dispersion analysis (FIDA)^24^ that allows the quantification of the strength of protein–(glyco)lipid vesicle binding, and on the other hand, we have used flow cytometry (FCM) to study the binding to (SA inhibitor-incubated) HEK-ACE2 cells by different domains of the spike protein to obtain a more detailed understanding of the molecular details of this biochemical process.

From these results, we infer that both the NTD and the RBD are able to bind SA moieties in a way that is beneficial for ACE2 binding. The MD simulations indicate that the NTD-SA binding is slightly stronger than the RBD-SA binding, the FIDA measurements experimentally validate the importance of the NTD-SA binding, and the FCM experiments reveal that the RBD-SA binding does not result in sequestering of its ACE2 binding site but instead leads to an increased ACE2 binding, given the strong SA ánd ACE2 dependence of the RBD binding to ACE2-transgenic HEK cells. The binding to SA moieties, omnipresent on the membranes of cells in the respiratory tract,^13^ allows the virus to diffuse on the surface of the cells. The subsequent binding to its main receptor, ACE2, is strongly enhanced by this two-receptor strategy.

## Methods

### Molecular Dynamics Simulations

The MD simulations are performed using GROMACS 2019.3.^25^ The topologies of the system are built using the CHARMM-27 force field.^26^ The selection of the CHARMM-27 force field for simulating protein-small molecule binding follows from its well-established reputation and compatibility with biomolecular systems.^27^ CHARMM-27 is specifically parametrized for proteins and nucleic acids, ensuring an accurate representation of their structural and dynamic behavior. Crucially, this force field includes comprehensive parameters for small molecules, providing a robust foundation for studying protein-small molecule interactions. Furthermore, the transferability of CHARMM force fields is advantageous, allowing for consistent and reliable simulations across various systems,^28^ e.g., systems containing (only or combinations of) lipids, proteins, nucleic acids, small molecules, etc. The extensive benchmarking and validation performed on the CHARMM-27 parameters^29^ provide confidence in the accuracy of the simulations. Finally, the force field is widely embraced in the MD simulation community, presenting the advantage of yielding easily interpretable and comparable results.

The protein is placed in a dodecahedron simulative box, with periodic boundary conditions, filled with TIP3P water molecules.^30^ For all simulated systems, we check that each atom of the proteins was at least at a distance of 1.1 nm from the box borders. Each system is then minimized with the steepest descent algorithm. Next, a relaxation of water molecules and thermalization of the system are run in *NVT* and *NPT* environments, each for a 0.1 ns at 2 fs time-step. The temperature is kept constant at 300 K with the v-rescale algorithm;^31^ and the final pressure is fixed at 1 bar with the Parrinello–Rahman algorithm.^32^ The LINCS algorithm^33^ is used to constrain hydrogen bonds. A cutoff of 12 Å is imposed for the evaluation of short-range nonbonded interactions and the Particle Mesh Ewald method^34^ is used for the long-range electrostatic interactions. The described procedure is used for all the performed simulations.

As the SARS-CoV-2 spike has 22 glycosylation sites, which play a crucial role in shaping the virus’s ability to infect specific cell types,^35,36^ we have checked if glycosylation might affect our 2D-Zernike and MD simulation results. However, none of the specific residues identified in our study as being involved in contact with SA are found in close proximity to a glycosylation site. Consequently, it is reasonable to leave out the glycan groups in our analyses, as the identified binding regions should retain the capacity to interact with SA, irrespective of glycosylation.

The full-length SARS-CoV-2 spike protein was simulated starting from the X-ray structure of the complex (PDB id: 6M17). We perform a 100 ns long simulation with a time step of 2 fs. The system is rendered electroneutral by adding 24 sodium counterions. The water density was set to 998 kg/m^3^.

To probe the NTD–SA interaction, the SARS-CoV-2 spike NTD (residue range: 16–290) is simulated in the presence of one molecule of SA (Neu5Ac) in solution. We select the domain ranging from residue 16 to 290 of the A chain of the trimeric complex. Likewise, the SARS-CoV-2 spike RBD (residue range: 331–524) is simulated in the presence of one molecule of SA (Neu5Ac) in solution. We selected this domain from residue 331–524. The same procedure is followed for the S1 domain (residue range: 1–700) of SARS-CoV-2 spike. All of these 3 simulations have been carried out for 1.75 μs with a time step of 2 fs.

### Energy Calculation

**Figure 2:**
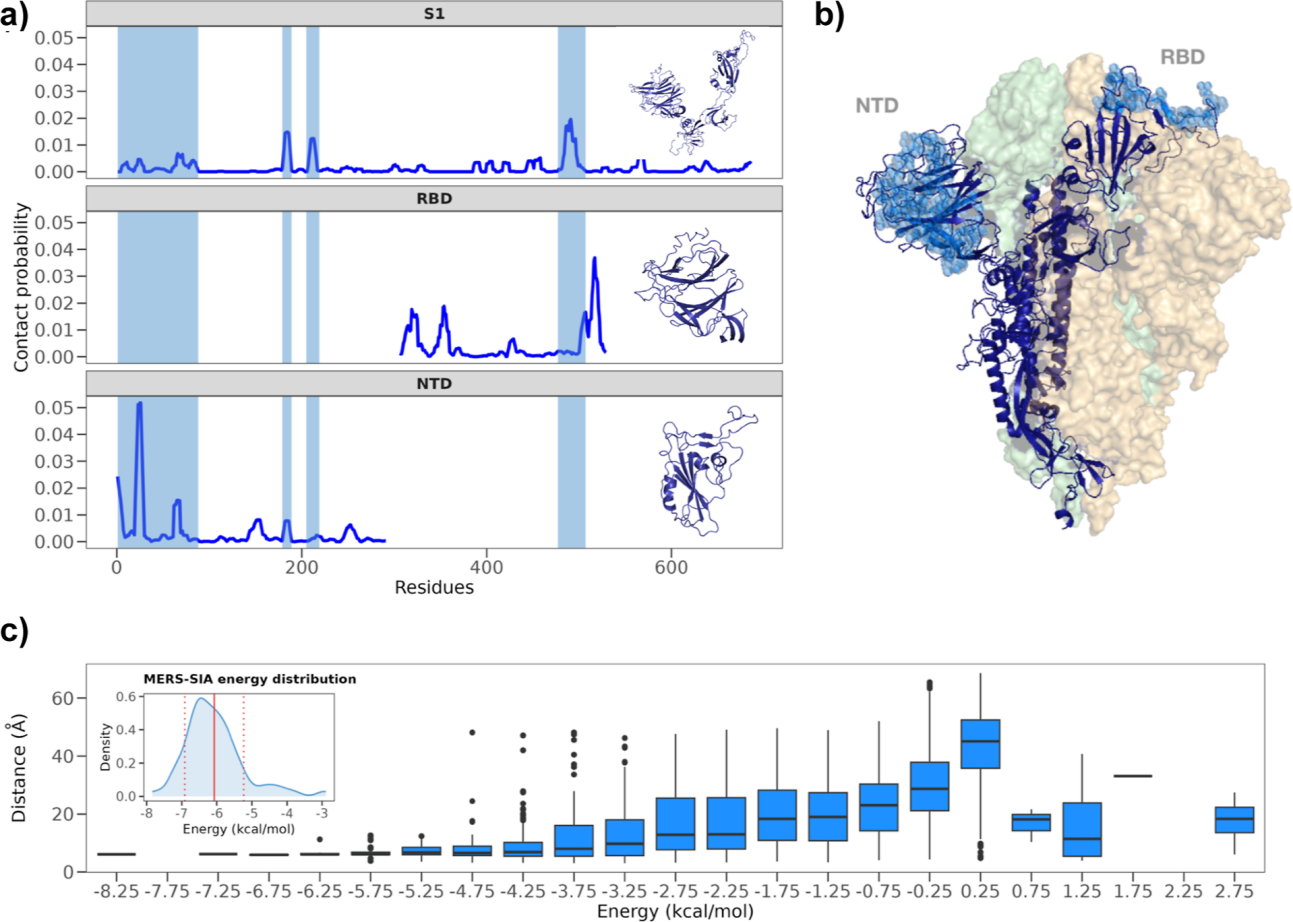
Contact probability as determined with MD simulations of the S1, RBD, and NTD domains of the SARS-CoV-2 spike. (a) Contact probability between each residue and the SA molecule is shown for the three molecular systems: the S1 segment, RBD, and NTD of the spike protein. (b) Molecular structure of the spike in its trimeric form: one chain is depicted as a blue-colored cartoon, and the molecular surfaces are highlighted for the remaining two chains. The residues with the highest probability of interaction, as calculated from the MD trajectory of the largest system, S1, are represented in cyan and are located both in the NTD and in the RBD. (c) Box-and-whisker plot of the minimum distance of the SA molecule to the nearest protein residue during the SARS-CoV-2 NTD MD simulation as a function of the binding energies. The inset displays the distribution of binding energies of the SA molecule to the MERS-CoV NTD during the simulation, with the thick red line indicating the mean value and the thin red line the standard deviation.

From the NTD simulation in complex with the SA (3 μs), we extracted a frame every 1 ns for a total of 3001 frames. On these frames, we calculated the energy using the Autodock software and the distance between the centroid of the Zernike pocket and the centroid of the SA. Then, placing ourselves in an interval that goes from the nearest integer less than the lowest energy found to the nearest integer greater than the maximum energy found, we grouped the energies at intervals of 0.5 kcal/mol and finally calculated the energy vs distance boxplot of Figure 2.

The insert on the MERS NTD binding energy distribution is instead calculated on the first 200 ns (201 frames, 1 ns step) of the MERS–NTD dynamics in complex with the SA.

In addition, to estimate the binding free energy between the spike protein and the SA molecule, we use the fastDHR tool, which is an open-access web server capable of predicting the binding free energy through methods based on MM/PB(GB)SA.^37^ More specifically, two binding poses extracted from the simulation of the NTD and SA were selected: the first corresponding to the minimum energy configuration as calculated through the Autodock method, and the second corresponding to the pose where SA is closest to the center of the pocket as predicted by the computational method based on the Zernike formalism.^38^ In this procedure, we selected the ff99SB force field (with TIP3P water model) for the receptor (spike protein) and the GAFF2 force field for the ligand (SA molecule).

### Flow-Induced Dispersion Analysis

FIDA experiments are based on the fact that the hydrodynamic radius of particles determines their laminar flow profile.^24,39^ By flowing mixtures of (glyco)lipid-containing vesicles and spike proteins through narrow (e.g., 75 μm diameter sized) capillaries, one will observe different flow profiles with versus without binding, because the bound complex has a larger hydrodynamic radius than the two unbound species. This effect is observed through fluorescent labeling of the spike proteins, recording the fluorescence intensity at a given point along the tubing as a function of time (see the FIDA figure in the Results Section for a graphical representation hereof), and plotting this in a so-called Taylorgram.^40^

#### Sample Preparation

The S1 segment (Gln14–Arg685) of the SARS-CoV-2 spike is expressed in modified human embryonic kidney (HEK) 293 cells by GenScript Biotech (NJ, USA), and the sequence and purity were checked by in-house mass spectrometry. The S1 segments of MERS-CoV (Tyr18–Pro747) and SARS-CoV (Ser14–Leu666) are also expressed in HEK293 cells, but by Bio-Techne Ltd. (UK). We dissolve the proteins in phosphate-buffered saline (PBS, Sigma-Aldrich, MO, USA) buffer (pH 7.4) and subsequently Alexa-488 label it in a nonspecific manner via amine coupling to the exposed lysine side chains (with a ∼50% efficiency). A stock solution of 2.4 μM S1-Alexa488 is prepared, which is subsequently diluted to a 100 nM concentration for the FIDA experiments.

SA (N-acetylneuraminic acid) with a >98% purity is purchased from CarboSynth (Compton, UK), and dissolved in PBS buffer and used at a 10 μM concentration during the experiment.

The lipid vesicles are prepared according to the protocol described in ref (41). The 1,2-dipalmitoyl-*sn*-glycero-3-phosphocholine (DPPC) and gangliosides GM1 and GM3 (extracted from ovine brains) lipids are purchased from Avanti Polar Lipids, Inc. (AL, USA), dissolved in chloroform and 50/50 vol % chloroform/methanol, respectively, dried under a N_2_ stream, and kept under light vacuum overnight. The next day, the lipids are resuspended in a PBS buffered H_2_O (milli-Q) solution, both as pure DPPC and at a GM1:DPPC and GM3:DPPC molar ratio of 1:9, with a total concentration of 1.3 mM. The solutions are then placed in a 60 °C water bath for 1 h and subsequently extruded 21 times through a 30 nm polycarbonate membrane with a mini-extruder system (both from Avanti Polar Lipids, Inc.) placed on a 60 °C heating block, which resulted in the formation of vesicles with a ∼50 nm diameter, as ascertained by dynamic light scattering (DLS), with a PDI of ∼0.15. The vesicles are then diluted to a 50–200 μM lipid (monomer)concentration for the FIDA experiments. In the concentration range 50–200 μM (corresponding to roughly 35–140 μg/mL), the volume fraction of lipids remains well below 0.1%, and we expect no major changes in solution viscosity over this range.

#### FIDA Experiment

To characterize the binding of the S1 segment of the SARS-CoV, MERS-CoV, and SARS-CoV-2 spikes to glycolipid-containing SUVs, we perform FIDA experiments using a FIDAlyzer instrument (Fida Biosystems ApS, Copenhagen, Denmark), with laser-induced fluorescence (LIF) detection (ZETALIF Evolution, Picometrics, Labege, France) with an excitation wavelength of 488 nm (Melles Griot Diode laser, Picometrics), in connection with an optical high-pass filter. The sample is flowed through a standard fused silica capillary (Fida Biosystems ApS) with an inner diameter of 75 μm, an outer diameter of 375 μm, and a total length of 100 cm with 84 cm between the sample reservoirs and the detection window. The capillary temperature is set to 25 °C inside the FIDAlyzer instrument, excluding the minor part connected to the LIF detector. Also, the capillary inlet and sample temperatures are heated to 25 °C.

The experimental protocol is then performed as follows (similar to ref (39)): first, the capillary is rinsed and equilibrated prior to each sample analysis with 1 M NaOH and PBS buffer at 3500 mbar for 45 s and 2 min, respectively. Then, the analyte sample (the vesicles) is injected at 3500 mbar for 20 s, after which the indicator sample (S1-Alexa488, mixed with the vesicles and the SA solution, with a preinjection incubation time of >10 min) is injected at 50 mbar for 10 s (39 nL, corresponding to 1% of the capillary volume). Finally, the injected indicator sample is then flowed toward the detection point with the vesicle sample at 50 mbar for 20 min.

All samples were performed in duplicate, and the Taylorgrams were processed using the FIDA data analysis software (Fida Biosystems ApS, Copenhagen, Denmark).

#### *K*_D_ Derivation

Because the FIDA data of the S1 spikes to the 1:9 GM3:DPPC vesicles does not have a sigmoidal shape, we estimate the dissociation constants (*K*_D_) by fitting the data with a normalized binding model, according to
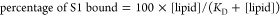
1

This model is based on a simple equlibrium between free and lipid-bound S1 and assumes (1) that at higher concentrations there will be a 100% occupancy, and (2) that the dose response curve has a standard slope equal to a Hill slope (or slope factor) of 1.0. All data could satisfactorily be fitted to a simple binding isotherm; there was no statistical basis for more complicated models, e.g., to include cooperativity.

### Flow Cytometry

FCM was employed to measure the binding of the various Alexa-488-labeled spike proteins to the ACE2 receptor. The HEK293 cell line stably expressing ACE2 was cultured in the presence of the Alexa-488-labeled spike proteins as described below, forming complexes upon binding. The fluorescence emitted from these complexes was then detected as the cells passed through a laser beam in a flow cytometer.

#### Gating Strategy

For the identification of the HEK-ACE2 cells, we first use the forward scatter area (FSC-A) vs the side scatter area (SSC-A) signals. The FSC-A and SSC-A refer to the measurement of the total light intensity in the forward and side scatter detectors, providing information about the sizes of the cells as they pass through the laser beam [see flow cytometry figure in the Results Section, panel (a)].

After this, we use the FSC-A plotted against the forward scatter height (FSC-H; the peak intensity) signals to select all the single cells within that HEK-ACE2 cell ensemble. This gating strategy facilitates the detection of single cells and discern them, for example, from events with multiple cells or cell debris [see flow cytometry figure in the Results Section, panel (b)].

The Alexa-488 fluorescence signal, which we record using the FL1-A channel for each event, is then directly proportional to the amount of labeled spike protein bound to each cell [see FCM figure in the Results Section, panel (c)]. The geometric mean of this fluorescence intensity (the gMFI) can thus be used to compare the binding under the various experimental circumstances.

#### Sialostatin Experiment

The HEK cells (HEK-293T) expressing human ACE2, HEK-293T-hACE2 Cell Line, and NR-52511, were obtained through BEI Resources, NIAID, NIH. The transformed cells were seeded at 50,000 cells/well in a 96-wells U-bottom suspension plate (CELLSTAR) and cultured in DMEM medium supplemented with 10% FBS, 1% l-glutamine, 1% antibiotic-antimitotic, and 1% l-pyruvate for 5 days with the addition of either 300 μM sialostatin or an equal volume of DMSO. At day 5, the cells were transferred to a 96-wells V-bottom plate and washed twice with PBS. Cells were subsequently stained in 25 μL/well of PBS supplemented with 0.5% BSA and 10 μg/mL of one of the following SARS-CoV-2-derived proteins or protein segments; spike glycoprotein (S), spike glycoprotein RBD, or spike glycoprotein domain S1 (S1) for 30 min at 4 °C. The S, S1, and RBD proteins (segments) were produced under HHSN272201400008C and obtained through BEI Resources, NIAID, NIH. The full length S protein from SARS-CoV-2 (Wuhan-Hu-1) has a C-terminal histidine tag, and was recombinantly expressed in HEK293F cells. It lacks the signal sequence and contains 1196 residues (the ectodomain) of the SARS-CoV-2 spike glycoprotein; the recombinant protein was modified to remove the polybasic S1S2 cleavage site (RRAR to A; residues 682 to 685), stabilized with a pair of mutations (K986P and V987P, wild-type numbering; GenPept: YP-009724390) and includes a thrombin cleavage site, T4 foldon trimerization domain and C-terminal hexa-histidine tag. The S1 segment was produced by transfection in HEK293 cells and purified. The S1 segment also lacks the signal sequence, contains 670 residues of the SARS-CoV-2 spike glycoprotein (amino acid residues V16 to R685), and features a C-terminal poly histidine tag. Finally, the RBD segment (also recombinantly expressed in HEK293F cells and featuring a C-terminal hexa-histidine tag) contains 223 residues (Arg319–Phe541) of the SARS-CoV-2 spike. Sialostatin or methyl 5-(ethylcarbamado)-2,4,7,8,9-penta-*O*-acetyl-3,5-dideoxy3-fluoro-d-glycero-β-galacto-non-2-ulopyranosonate is an efficient metabolic inhibitor of sialyltransferases and was synthesized from commercially available SA, as previously reported (see ref (42) for the most recently reported EC_50_ values and updated synthesis; it was first reported in ref (43)).

After incubation, the cells were washed twice with PBS supplemented with 0.5% BSA, followed by a primary antibody or probe staining for 30 min at 4 °C with either rabbit anti-his tag antibody (Abcam, ab14923, 1:90), goat anti-ACE2 antibody (R&D Systems, AF933, 1:400), or SiaFind Pan-Specific Lectenz (LectenzBio, 1:400). Subsequently, two washes with PBS and 0.5% BSA were followed by the addition of PBS with 0.5% BSA and either donkey-anti-rabbit IgG (H&L) Alexa 488 (Thermo Scientific, A21206, 1:400), donkey-antigoat-IgG (H&L) Alexa 488 (Thermo Scientific, A11055, 1:400) or Streptavidin-488 (Thermo Scientific, S32354, 1:1000). The secondary antibody staining was incubated for 10 min at 4 °C. Prior to analysis on a Cytoflex S flow cytometer (Beckman Coulter), the cells were washed twice with PBS with 0.5% BSA and resuspended in PBS.

#### HSase Experiment

The binding of the SARS-CoV-2 S, S1, and RBD to HEK-ACE2 cells upon the addition of HSase was assessed using FCM. In short, 100,000 HEK-ACE2 cells were seeded into a 96-wells plate and incubated with or without the addition of HSase mix (2.5 mU/mL HSase I, 2.5 mU/mL HSase II, and 5 mU/mL HSase III; obtained from IBEX Pharmaceuticals, Inc.). The cells were subsequently stained with 20 μg/mL of the his-tagged SARS-CoV-2 S, S1, and RBD proteins (vide supra) in PBS with 0.5% BSA. After washing the cells, the cells were stained with either rabbit anti-his tag antibody (Abcam, ab14923, 1:90) or HS4C3 anti-HS antibody (used 1:10 from aliquots). The secondary antibody staining was done using donkey-anti-rabbit IgG (H&L) Alexa 488 and P5D4 mouse anti-his tag antibody (used 1:10 from aliquots). As a tertiary antibody, goat-anti-mouse IgG—Alexa-fluor488 (Fisher Scientific catalog #: 10256302) was used. Before analysis on a Cytoflex S flow cytometer (Beckman Coulter), the cells were washed twice and resuspended in PBS.

## Results

### Quantification and Prediction of the Binding between SARS-CoV-2 Spike and SA Using MD Simulations

In an earlier computational investigation,^19^ we compared the structures of the NTD of MERS-CoV, SARS-CoV, and SARS-CoV-2 spike proteins by evaluating their similarity using a computational method that relies on so-called 2D-Zernike polynomials. This methodology allows a structural comparison of different molecular regions and the highlighting of conformational differences and similarities. Based on our findings, we identified a binding region in the NTD of the SARS-CoV-2 spike for SA molecules. In that analysis, the SARS-CoV NTD was employed as a negative control, as this virus lacks SA binding capabilities.^7^ In Figure 1a–c, we show the surface of the NTD region in the three different spikes, with the region that has a high similarity with the MERS-CoV region colored in red. Notably, the finding that SARS-CoV-2 has SA binding capabilities was subsequently experimentally validated,^20,44^ thus confirming the predictive power of the 2D-Zernike method to recognize binding pockets (see Figure 1d–f).

Here, we extend our analysis to the whole S1 region of the spike, which contains the NTD and the RBD using MD simulations, allowing us to compare the SA binding capabilities of different spike domains. Specifically, we performed MD simulations on the full S1 segment, the RBD, and the NTD, in each case, together with one molecule of SA in explicit water. No additional potential was included, so that the SA molecule was free to diffuse in the aqueous environment and interact with the protein domain.

Recent cryo-electron microscopy experiments have shown that the structure of the separate domains of the spike protein is conserved when they are isolated from the full trimeric complex.^45^ In order to determine if the dynamics of the isolated domains are the same as in the full-length trimeric spike and to avoid simulating the entire spike trimeric complex, thereby speeding up the simulations, we have first performed a simulation of the various segments and the full-length and trimeric SARS-CoV-2 spike. These simulations confirm that the RBDs, NTDs, and S1 segments have similar structure and dynamics in their isolated and trimer-incorporated forms (see Supporting Information, Figure S1). The analysis indicates that the behavior of each NTD, RBD, and S1 segment of the trimer system is comparable with the domain simulated alone in solution, both in terms of root-mean-square deviation (rmsd) and root-mean-square fluctuation (RMSF) descriptors. In particular, the comparison between the RMSF of the domains considered alone vs in the trimer shows that the most and least fluctuating regions are conserved. The average Pearson correlation between the trend of the RMSF of the three domains of the trimer with the RMSF of the NTD of chain A alone is 0.63. Taken together, these results allow us to simulate only the NTD, RBD, and S1 of the SARS-CoV-2 spike protein, thus significantly reducing the computational cost of our simulations.

Furthermore, simulating S1 allowed us to analyze the correlated motions among residues of both the RBD and NTD of the spike protein. The analysis of correlated motions among residues, both considering covariance (to assess the average fluctuation of each residue) and considering the Pearson coefficient (which is independent of the fluctuation of individual residues), provides insight into communication among different regions that may play a key role in function and structural dynamics.^17,46,47^ We examine both Pearson correlation and covariance between residues (averaging over *x*, *y*, and *z* correlation values for each residue pair). Specifically, we focused on the cross-correlation between the NTD and RBD domains of the S1, studying the correlated movements both within the domain (i.e., among residues belonging to the same domains; NTD–NTD and RBD–RBD) and between domains (i.e., among residues belonging to two different domains, NTD–RBD).

Interestingly, we observe a positive Pearson correlation for residue pairs within the same domain, both NTD and RBD, with average values of 0.056 and 0.043 for residues belonging to the NTD and RBD, respectively (see Supporting Information, Figure S2). Conversely, for residues belonging to the two different domains (NTD and RBD), we note a significant anticorrelation (negative correlation) with a Pearson coefficient of −0.042. Although analyses of correlated motions are widely used for analyzing molecular simulation data, the interpretation of average intra- and interdomain correlations is not straightforward. The positive correlation between nearby residues within the same domain may likely determine the conservation of binding regions, both directly and indirectly. Conversely, the correlation (in this case negative) between residues belonging to the two domains could quantify how the two domains communicate with each other, probably to maintain (or alternate) binding propensity.

To better understand the mechanisms of synchronized motion between residues of the two domains (NTD and RBD), we selected the set of residues that maximizes the anticorrelation between the two domains (see also Figure S2). A set of residue–residue pairs was chosen, which exhibited anticorrelation values ranging from −0.33 to −0.38. Specifically, the residues range from 182 to 187 in the NTD and from 386 to 391 in the RBD. In both cases, the residues belong to loop regions. Interestingly, we identify two regions that do not overlap with the SA binding regions but which could play an important role in the dynamic-structural relationship between the two domains.

Next, we performed MD simulations of the NTD, RBD, and S1 domains of the SARS-CoV-2 spikes in the presence of a SA molecule to observe events of interaction. To study the interaction between the protein and SA, we first defined a dynamic binding propensity score for each residue of the simulated domains, which is based on the time each residue spends in interaction with SA during the evolution of the simulation. For each residue, we calculated the fraction of frames in which the centroid atom of the SA molecule is located at a distance less than 6 Å to at least one of the heavy atoms of the residue (see Figure 2a). This allows us to compare the SA contact probability per residue obtained from MD simulation of the various SARS-CoV-2 spike domains with the results from the Zernike shape complementarity evaluation,^19^ through the binding propensity derived from the MD trajectory.

To avoid spurious contacts, i.e., contacts due to the limited size of the simulation box, we considered only configurations having favorable energies. To this end, we first estimated the binding energy between the NTD of MERS-CoV spike and SA by performing a MD simulation starting from the experimental pose^12^ (see Methods). More specifically, sampling configurations of the system at the equilibrium (see Methods for details), we calculated the intermolecular energy contribution using the AutoDock^48^ for each selected pose. The distribution of these energies is reported in the inset in Figure 2c. Despite the differences between these two systems, the energy distribution of the interaction between the MERS-CoV spike protein and the SA molecule allows us to energetically relate the interaction between SA molecules and the SARS-CoV-2 spike with what we would expect experimentally, under very similar conditions.

We then extracted the interacting conformations and calculated the intermolecular energy using the AutoDock algorithm for each of them: we thus selected only the poses characterized by an intermolecular energy lower than −4 kcal/mol. As further validation, we studied the intermolecular interaction between SA and SARS-CoV-2 spike protein as a function of the distance between SA and the putative binding region predicted with the Zernike-based approach. As is evident from Figure 2c, when the SA molecule is close to the predicted region, its interaction energies are favorable, while higher energies characterize the poses where the SA molecule is far from the predicted binding site. This result confirms that the preferred spike region of interaction is the one with the highest binding compatibility, as calculated by our Zernike method.

To further test the revealed binding region, we sought for an evaluation of binding free energy via fastDRH^37^ (freely available as a web server), which provides a MM/PB(GB)SA-based free energy estimation given a certain binding pose. In particular, we selected two binding configurations between the spike protein (S1) of SARS-CoV-2 and the SA molecule. The first configuration corresponds to the complex for which the binding energy, estimated with Autodock,^48^ is minimal compared to all other frames (−8.2 kcal/mol). In this configuration, the SA molecule is located at a distance of approximately 6 Å from the center of the binding pocket, as predicted computationally by the Zernike polynomial-based method. The second selected configuration is the one in which the intermolecular energy between the spike and the SA molecule is minimal among the frames in which SA binds to the pocket predicted by the Zernike-based method. In this way, we selected the structure with the minimal energy of the spike-SA complex, in which SA binds to the predicted pocket. Calculating the free energy with MM/PB(GB)SA methods for both selected configurations, we confirmed the estimated energy value for the first configuration (obtaining a value of −8.22 kcal/mol with Autodock and an average value of −8.61 kcal/mol with MM/PB(GB)SA), on the other hand, we got a considerably more favorable (negative) free energy value for the binding between SA and the pocket predicted with Zernike when MM/PB(GB)SA methods are used. Specifically, for this configuration, the energy calculated with Autodock is −4.99 kcal/mol (indicating a good interaction energy, among the best explored blindly in the simulation), while the energy calculated with MM/PB(GB)SA is −12.10 kcal/mol. This shows that the pocket predicted with the Zernike method could bind to the SA molecule even more favorably than the possible neighboring binding sites. The comparison between the estimates obtained with Autodock and those obtained with fastDRH is reported in the Table S1 in the Supporting Information.

Interestingly, looking at Figure 2a, not all the peaks in the S1 simulation match with single-domain simulations. This suggests that some binding regions of the single domains could be in nonphysical regions; in other words, in a region of the sequence that faces the inner part of the fully folded spike trimer that cannot interact with other molecules in the biological context. To elucidate this, in Figure 2b, the full trimer is depicted with one S1 chain highlighted in blue, and the solvent-accessible regions of the electrostatic surface of the RBD and NTD that have a high probability of interaction with SA are marked in cyan. To compute the regions that are solvent-exposed in the trimer, we made use of the short MD simulation of the full trimer (vide supra). For the RBD, this excludes the region from residues 320 to 380. During the SA-RBD simulation, 23.3% of the SA binding time occurs to RBD residues that are not accessible to SA molecules in the fully folded spike protein. The identification of nonbiologically relevant (i.e., regions that are not solvent exposed in the full-length, folded protein) binding sites indicates that the RBD is not a proper model protein to study SA binding by the S1 segment or full-length spike.

To quantify which binding is stronger and more physically significant, we computed the relative time that the SA molecule spends in the NTD and in the RBD binding pocket in the S1 MD simulations. By integrating over the contact probability and determining the ratio among these times, we can estimate which is the stronger binder among the two domains. We find that the NTD spends 0.211 ms bound, against 0.167 ms for the RBD. Since the binding probability is intrinsically proportional to the time spent bound,^49^ and because these two residence times are computed from the same simulation, we can speculate that the SA binding strength of the NTD is around 25% stronger than the SA binding strength of the RBD. From this, we conclude that NTD and RBD binding to SA moieties have an approximately equally strong, positive effect on the probability of infection of SARS-CoV-2.

### Flow-Induced Dispersion Analysis Shows That SARS-CoV-2 Spike Binds to Sialic Acid *In Vitro*

**Figure 3:**
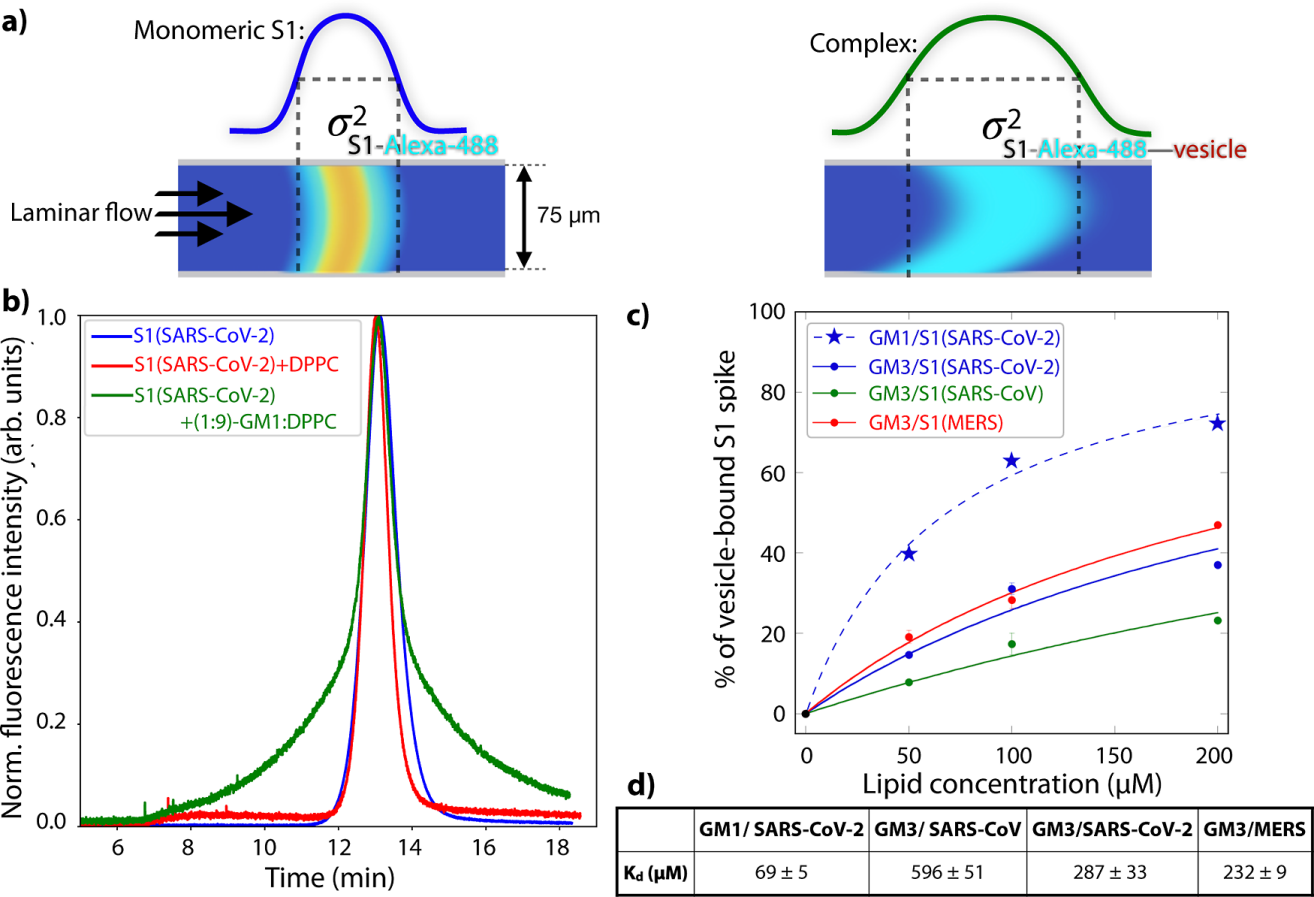
FIDA of SARS-CoV-2 spike S1 segment binding to SA containing glycolipids. (a) Schematic of how complex formation by fluorescently labeled S1 will affect the Taylorgrams of FIDA measurements. A single species that includes a fluorescent label will give rise to a single Gaussian line shape, with a Gaussian width σ that increases with an increasing hydrodynamic radius. (b) Taylorgrams recorded for pure 100 nM spike S1 protein, with 200 μM DPPC vesicles, and with 1:9 GM1:DPPC vesicles, partly composed of the SA containing GM1 glycolipid. In the case of the glycolipid-containing vesicles, a double Gaussian line shape is observed, indicating the presence of both free S1 species and S1-vesicle complexes, as opposed to the single Gaussian lineshapes observed for the pure S1 for in the presence of the DPPC vesicles that do not contain glycolipids, indicating the absence of S1-binding in that case. (c) The relative number of bound species for the S1 segments of SARS-CoV, SARS-CoV-2, and MERS-CoV bound to (1:9)-GM1:DPPC and -GM3:DPPC lipid vesicles (lipid concentration in monomer units). Lines show best fit to eq 1. (d) Estimated dissociation constants (K_D_s).

Our *in silico* findings predict that the S1 segment of the SARS-CoV-2 spike protein is able to bind to SA moieties.^19^ To experimentally confirm these predictions, we perform FIDA measurements^24,39^ with the S1 segment of the protein (residues Val16–Arg685, see GenBank accession number GenBank: QHD43416.1^50,51^ for the full sequence), recombinantly expressed in human cells (see Methods). The FIDA technique relies on the fact that particles with a larger hydrodynamic radius (*R*_h_) will be dispersed more strongly in a laminar flow than particles with a small *R*_h_ (see Figure 3a). By labeling the S1 spike segment nonspecifically with a fluorescent dye and flowing it through 75 μm diameter capillaries in the presence and absence of vesicles partly composed of SA containing glycolipids, the observed *R*_h_ is expected to strongly increase upon S1–glycolipid binding. The S/N of the FIDA setup is not sufficient to detect complex formation between the S1 spike and free SA, but the binding to glycolipid-containing vesicles can be characterized well. The mechanistic nature of this experimental setup allows us to determine if SA can play a physical role in SARS-CoV-2 infection in a cellular context. Indeed, if the SARS-CoV-2 S1 segment can bind SA-containing vesicles, the physical strength of this binding can be considered relevant in a cell–virion context.

When we compare the Taylorgrams of a pure S1 solution with those of either pure DPPC ∼50 nm small-unilaminar vesicles (SUVs), or with 1:9 GM1/DPPC composed SUVs, we observe the *R*_h_ of monomeric S1 if there are no glycolipids present in the system, but if there are glycolipid-containing vesicles, the increased *R*_h_ indicates complex formation. This result is corroborated by previously published findings for SA molecules that were connected with 50-mer PHEA polymer chains to Au nanoparticles, which also exhibited SARS-CoV-2 spike binding.^44^ Our experiments are performed in the presence of 10 μM free SA, to prevent nonspecific binding between the spike S1 protein and DPPC, as evinced by sharp peaks in the Taylorgrams (see Figure S3). While pure monomeric S1 and S1 in the presence of SUVs composed solely of DPPC give a unimodal Gaussian line shape of the Taylorgrams, the S1 binding to 1:9 GM1:DPPC vesicles results in a two-modal Gaussian line shape, which (through fitting, see ref (24)) indicates that approximately 50% of the S1 spikes are bound to the vesicles, while the other ∼50% remain unbound.

To investigate if the Zernike analysis also correctly predicts the absence of this SA binding pocket in the S1 segment of SARS-CoV,^19^ while it is also present in MERS, and to determine if the binding is dependent on the type of glycolipid, we repeated the experiment by incubating 100 nM of each of the three S1 segments, but now in the presence of varying amounts of 1:9 GM3:DPPC lipid vesicles. The glycolipid is varied from GM1 to GM3 to investigate if the exact position of the SA group within the sugar chain in the lipid headgroup^16^ affects the binding. The FIDA measurements show (1) that the SARS-CoV-2 and MERS-CoV S1 segments bind ∼ twice as strong than the SARS-CoV S1 segment, as evinced by the ∼ twice as high percentage of vesicle-bound S1 at a given lipid concentration, and (2) that the SARS-CoV-2 spike binds stronger to GM1 than to GM3 (Figures 3c and S4 for the associated Taylorgrams). In order to obtain an estimation of the dissociation constant K_D_, even though no saturation is obtained before vesicle clustering occurred during the FIDA measurement, we assume that at higher lipid concentrations, 100% occupancy would have been reached. Under this assumption, we find apparent K_D_s that are (1) ∼twice as small for SARS-CoV-2 and MERS as compared to SARS-CoV-1, and (2) ∼four times as small for SARS-CoV-2 spike binding to GM1 as compared to GM3 (see Figure 3d). The latter observation indicates that the extra sugar groups present in the headgroup of GM1 (absent in the GM3 headgroup) mediate the spike-SA binding. Furthermore, we think that the nonzero SARS-CoV S1 binding to the glycolipids is due to nonspecific interactions, and we note that—given the omnipresence of SA moieties on cell membranes—even the relatively weak SA binding revealed here may suffice to greatly enhance the infection efficiency.

### Flow Cytometry Shows That SARS-CoV-2 Spike Binds to SA Moieties on Cell Membranes

**Figure 4:**
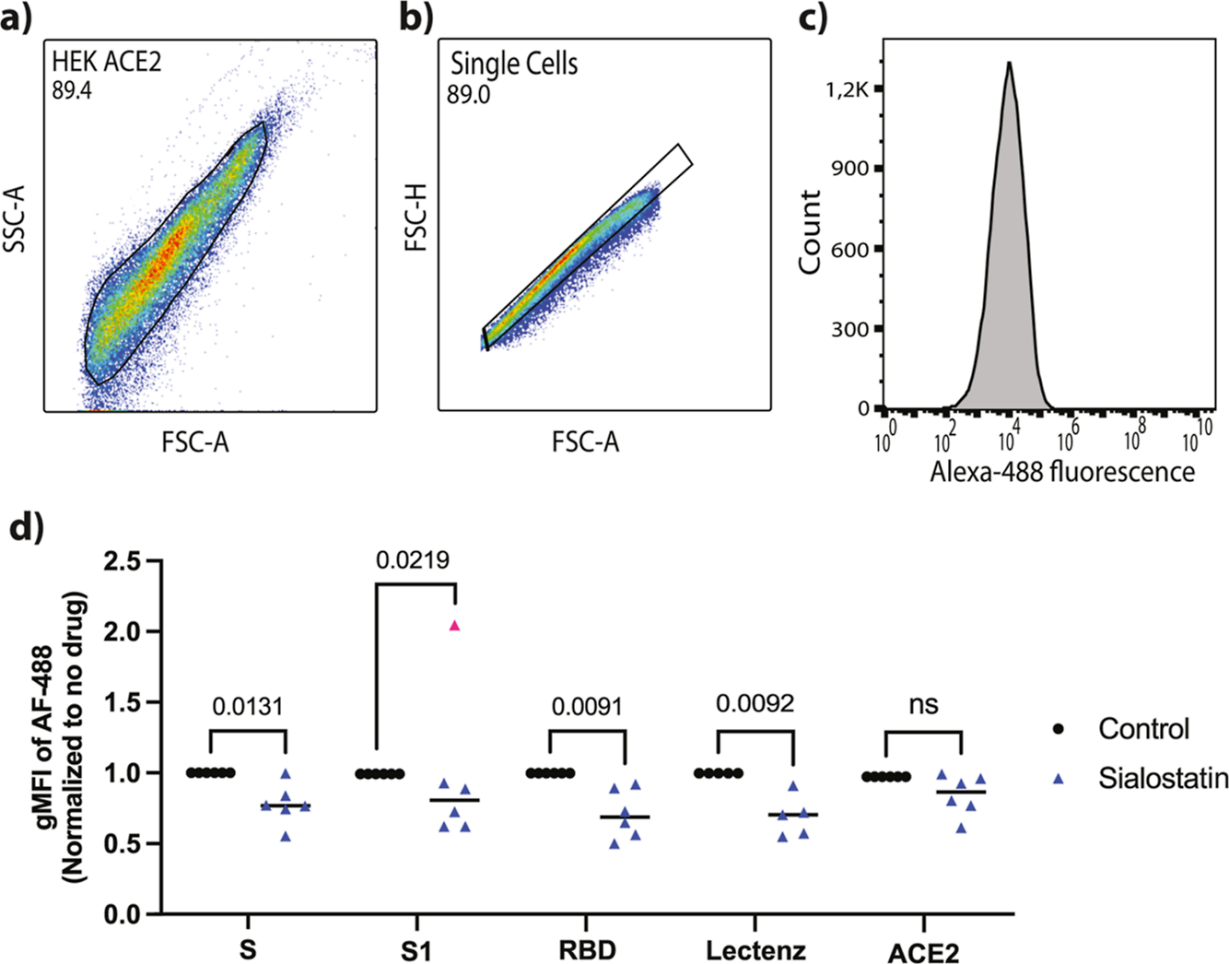
Decreased binding of the SARS-CoV-2 full-length spike, S1, and RBD proteins to HEK-ACE2 cells upon inhibition of SA expression. (a–c) Example of an applied FCM gating strategy to assess full-length spike protein binding to single HEK-ACE2 cells, and the Alexa-488 fluorescence resulting from spike binding to the gated ensemble. (d) The normalized geometric mean of the Alexa-488 fluorescence intensity (gMFI) that indicates the relative binding strength of various segments of the SARS-CoV-2 spike protein to HEK-ACE2 cells, the Lectenz binding (which reports on the presence of SA groups on the cellular membrane), and ACE2 levels, as determined by FCM, with and without the addition of sialostatin (*n* = 6, 2-sided paired *t*-test). Statistics per experiment: SARS-CoV-2 spike S1 domain: (*n* = 5, 2-sided paired *t*-test), with one outlier (marked pink) was excluded based on a GRUBS test (alpha 0.05); SARS-CoV-2 spike RBD domain: *n* = 6, 2-sided paired *t*-test; SiaFind Pan-Specific Lectenz with and without the addition of sialostatin: *n* = 5, 2-sided paired *t*-test; ACE2 expression level of HEK-ACE2 cells: (*n* = 6, 2-sided paired *t*-test). ns: not significant.

The *in silico* 2D-Zernike analyses and MD simulations predict that the spike S1 can bind SA through different domains, while the FIDA measurements show the physical relevance of the structural variation among various β-coronavirus spike NTDs in a lipid-membrane context. In order to see if these indications also stand in the spike interaction with cells, we perform FCM measurements. We do this by determining the binding of three different spike segments to transgenic HEK cells that are transfected to express a large number of ACE2 receptors. First, we investigate the full-length spike protein (Cys-15–Pro-1213)—given that this has the closest similarity to the spike protein attached to the SARS-CoV-2 virus, the S1 segment (Val-16–Arg-685)—given that it contains both SA binding regions of the SARS-CoV-2 spike protein, and the RBD region (Arg-319–Phe-541)—given the unexpected result obtained in the MD simulations that the ACE2 binding site (which gives the RBD its name) also has SA binding capabilities. To reveal the influence of cell-membrane associated SA groups on the binding, we add a SA expression inhibitor called sialostatin (see Methods for details). When we gate the FCM such that the Alexa-488 fluorescence reflects the spike-binding events to the HEK-ACE2 cells (see Figure 4a–c), a significant decrease in the binding of each of the three investigated domains (the full-length spike, the S1 domain, and the RBD; see Figure 4d) occurs when the HEK-ACE2 cells are incubated with sialostatin. The fact that the binding of all of the investigated domains decreases is consistent with the MD simulations that indicated SA binding capabilities in the three segments.

As can be seen from the SiaFind Pan-Specific Lectenz binding, which reports on the level of SA on the cell membrane, the degree of sialostain-induced signal decrease is similar for the three spike domains as for Lectenz, while the ACE2 levels are not affected. This indicates that the availability of SA groups on the membranes of HEK-ACE2 cells very strongly affects the ability of the three spike domains to bind and that there is no significant contribution to the spike-cell binding through direct ACE2 binding.

To further disentangle the relative roles of the SA and ACE2 receptors in the spike (segment) binding, we also performed FCM measurements with wild-type HEK cells (Figure S5). Instead of removing SA moieties from the cell membrane, as in the experiment shown in Figure 4, we thus observe the effect of removing ACE2 groups. A comparison of the spike binding to transgenic ACE2-HEK cells and to wt-HEK cells reveals a strongly decreased binding to the latter. The two FCM experiments thus show that the cellular binding of the spike segments is dependent on the presence of both SA and ACE2 receptors. We can thus derive that the initial spike–SA binding, which is of vital importance to the initial spike–cell binding according to the results depicted in Figure 4—does not suffice by itself to maintain the spike-cell binding at a similar level as for the HEK cells that have a higher ACE2 expression. We think that the origin of the decreased binding as a function of decreased SA expression (Figure 4) instead lies in a decreased initial molecular recognition between the spike and SARS-CoV-2 host cell. Potentially, if larger decreases in SA expression could be obtained in future studies, the relative importance of the two receptors could be explored in more detail. As we now see maximal inhibition of the binding (1:1) for the achieved inhibition of SA expression and also a very strong decrease in the spike binding without enhanced ACE2 expression (Figure S5), it is probable that the importance of both receptors for successful binding is comparable in magnitude.

As we have incubated the cells with a single concentration of spike (domain) proteins, we cannot determine a binding strength, but the FCM measurements nonetheless clearly confirm the biological relevance of the most important computational predictions, namely, that SARS-CoV-2 exhibits a two-receptor strategy and that the RBD is also strongly involved in SA binding, which has a positive contribution to eventual ACE2 binding.

Overall, the FCM experiments demonstrate the biological relevance of the SA—spike (segment) binding observed with the *in silico* and FIDA methods at a cellular level.

## Discussion

### Molecular Model of SARS-CoV-2 Binding to SA

By closely integrating molecular simulations and specific experimental binding assays, we have investigated the interaction between the spike protein of SARS-CoV-2 and SA. In this research, we have used computational methods to first discover SA as a previously unknown binding partner for the SARS-CoV-2 spike protein (with the Zernike approach^19^), after which we have identified two binding regions on the spike (with the MD simulations—see Figures 1 and 2). To validate these findings, we have first confirmed the specific SARS-CoV-2–SA binding in the well-defined model system of SA containing lipid vesicles and the spike S1 segment that contains both SA binding sites (with FIDA—see Figure 3), and subsequently confirmed a similarly specific binding to ACE2-expressing cells for various spike segments to show that also in a biologically relevant and complex setting this is a relevant process (with FCM—see Figure 4).

The main finding from this combination of methods is the observation that within the S1 segment of the spike protein there are two regions that interact with SA groups, namely, the NTD and the RBD. So far, there is not a well-known biological role of the NTD for the entry of the viral RNA inside the cell, while the RBD is known to bind the principal SARS-CoV-2 receptor ACE2, which leads to virion internalization.^52^ For MERS-CoV, the binding pocket of SA is known to be an anchor for its spike to bind the cell membrane as a first step in the infection mechanism.^12^ Here, we show that the NTD plays a similarly critical role in the infection mechanism for SARS-CoV-2. Based on the experimental and *in silico* evidence presented in this work, it appears that the SARS-CoV-2 spike first binds SA moieties on the cell membrane, after which the spike-SA complex diffuses on the membrane to find the ACE2 receptor.^53^ The combination of the 3D diffusion, to reach the cell, and 2D diffusion to search across the membrane, strongly increases the residence time on the membrane during which it can meet its principal receptor. We note that quantification is difficult because of the large number of physical constants (relative concentrations, exact *in vivo* binding constants, etc.). Building a molecular model that fully describes all the relevant interactions cannot be achieved by experiments or simulations alone.

We therefore performed experiments at different biologically relevant levels and complementary MD simulations to obtain a molecular-level interpretation. Indeed, on the one hand, based on FIDA, we are able to show that, at an *in vitro* scale, the S1 segment of the SARS-CoV-2 spike is able to bind lipid vesicles that are partly composed of SA containing glycolipids. We observe that this binding was much stronger for the MERS-CoV and SARS-CoV-2 S1 proteins, whose NTD has been shown to have a similarly shaped SA binding pocket,^19,54^ as compared to SARS-CoV, for which such a similarly shaped region was found to be absent in the Zernike analysis. This substantiates the importance of the role of the NTD in the SA–SARS-CoV-2 spike binding and corroborates recent experiments that directly reveal SARS-CoV-2 spike NTD–SA binding.^55,56^ On the other hand, we also performed FCM measurements to show that the spike-SA interaction is essential in the cellular binding probability by incubating the cells with the SA inhibitor sialostatin. By assessing this binding for various spike segments, we find a similar SA inhibition effect on the cellular binding of the RBD, the S1, and the full-length spike, consistent with the MD findings that each of these segments contains SA binding sites.

The MD simulations also provide a molecular-level image of what happens during the binding experiments. By analyzing the structure of the RBD, NTD, and S1 segment, either as a part of the fully folded spike protein or as a separate domain, we were able to reproduce the NTD binding pocket that was previously found with the Zernike analysis^19^ and show that the experimentally observed SA binding of the isolated RBD is to a certain degree biologically irrelevant as ∼25% of the SA binding residues are not accessible to SA in the fully folded spike protein. From the SA–S1MD trajectory, we have determined the respective SA affinities to the NTD and RBD domains. Using a simple dynamical model and deriving physical constants from the MD simulations, we are able to estimate the effects of the two domains on SA binding, which—as a secondary receptor—helps the viral infection. This indicated an additional ∼25% stronger binding of SA to the NTD than to the RBD.

Interestingly, we observe SA—, but not heparan-sulfate (HS)—binding in both the FIDA (for the S1) and (for neither the RBD, S1, nor full-length S segments) FCM experiments (see Figure S6), while a previous FCM study has reported RBD and S binding to, e.g., *Vero E6* and *A549* cells,^16^ a fluorescent microarray and surface-plasmon resonance study has reported RBD and S binding to HS, but not to SA,^59^ and a NMR study has reported that S, but not RBD binds to α2,3- and α22,6-sialyl *N*-acetyllactosamine, i.e., two SA containing trisaccharides.^55^ In these FCM experiments, performed with HSases (instead of sialostatin), we observed strongly decreased HS levels on the HEK-ACE2 cells but not any decrease in spike (segment) binding, as opposed to earlier findings for different cell lines by Clausen et al.^16^ Also, in FIDA experiments, we observe no RBD or S1 binding to heparin of various molecular weights (6–30 kDa) nor to the HS-chain containing proteoglycan syndecan-2 (data not shown), while this was observed for immobilized HS,^60,61^ and—in a typically unspecific manner—for heparin.^62−65^ Taken together, this indicates that the exact choice of molecules, cells, and techniques significantly influences the outcome of these experiments.

## Conclusions

**Figure 5:**
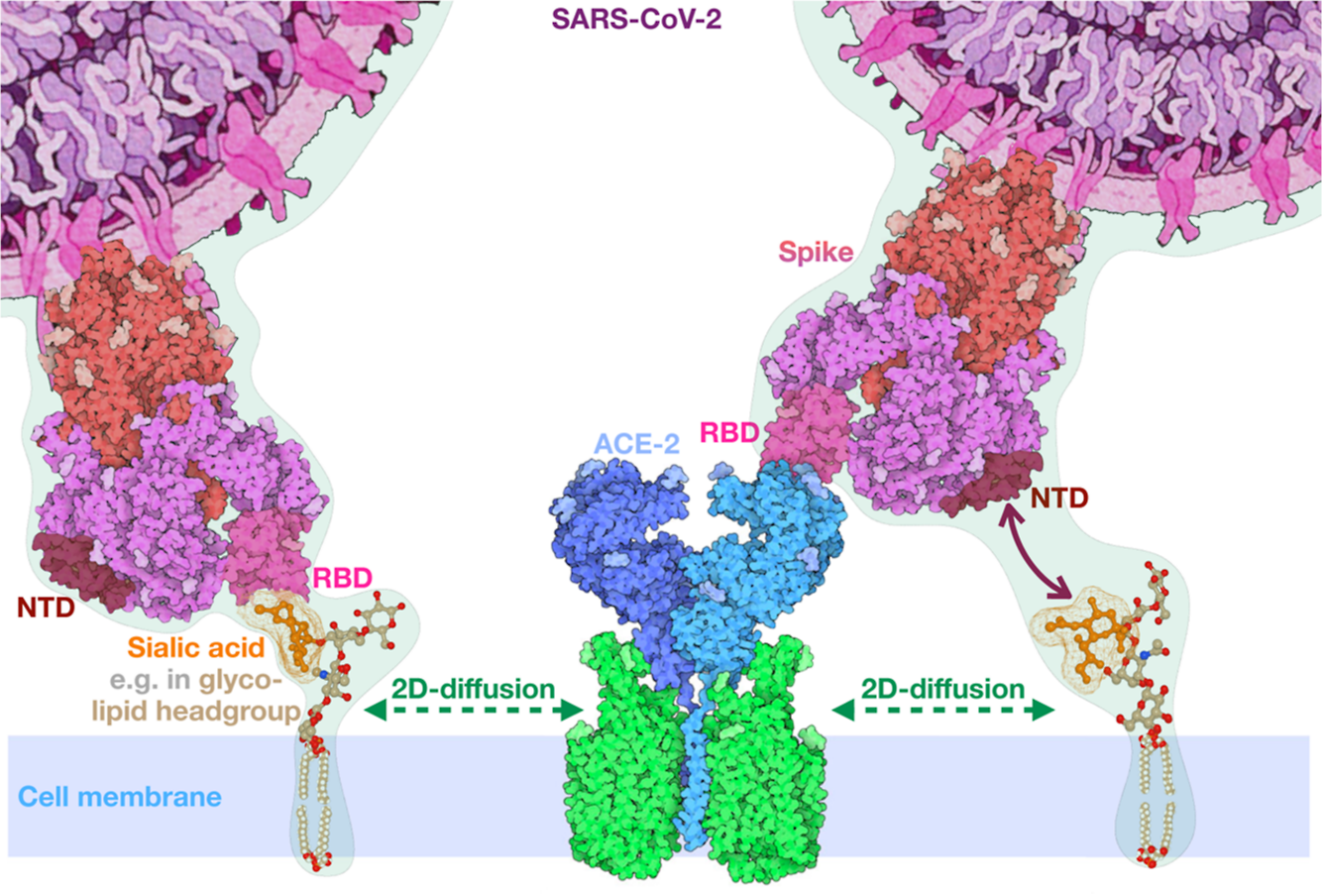
Two receptor binding strategy of SARS-CoV-2 is mediated by both the N-terminal and receptor-binding spike domain. Computations and experiments reveal that both the NTD and RBD can bind to SA groups at the cell membrane. Both binding events have a positive effect on subsequent ACE2 binding. After a 3D diffusion process in which the spike proteins can interact with the omnipresent SA moieties on cellular membranes, SA binding will reduce the dimensionality of the search for the ACE2 to a 2D-diffusion process. As the latter binding triggers virion internalization,^52^ the two-receptor and two binding-domain strategy of SARS-CoV-2 strongly enhances its infection rate. Protein and virion renderings are reproduced from ref (57) under the CC-BY-4.0 license and the lipid rendering is created in *UCSF Chimera*.^58^

The observed SA binding by the SARS-CoV-2 spike could have profound consequences in the context of the human pandemic situation. Indeed, closely related species of β-coronaviruses, such as MERS-CoV and SARS-CoV, have developed different strategies to infect the host cells. SARS-CoV utilizes the ACE2 receptor with a high affinity, and binding ACE2 with its spike protein starts the chain of chemical reactions that leads to the insertion of the viral RNA. In a different fashion, the MERS coronavirus uses SA moieties on the cell membrane as the main attachment receptor, after which it searches for its main receptor, DPP4, on the membrane. Here, we show that SARS-CoV-2 has developed a different infection mechanism compared to these other coronaviruses (see Figure 5). On the one hand—as is the case for SARS-CoV infection—the SARS-CoV-2 virion internalizes after binding to the ACE2 receptor.^52^ On the other hand—as is the case for MERS-CoV—it also has an affinity for SA, which is used as a first anchor to engage the virus to the host cell. Two-dimensional diffusion on the cell membrane after SA binding strongly accelerates the search for SARS-CoV-2’s internalization receptor, ACE2. In light of these observations, we propose the possibility that SARS-CoV-2 uses a combined strategy. Such a double-receptor strategy, in which two spike domains are both able to bind the SA moieties on the cellular membrane in the initial step, could explain the very fast spread of this virus.

One of the main problems with regards to the spreading of this virus has been the very high number of asymptomatic patients that have spread the virus without being aware of carrying it. It is possible that this observation can be linked to the here-described SA binding (although our FCM experiments show very limited spike–cell binding in the absence of ACE2). But SA has been found to be omnipresent on the membranes of cells of the external respiratory airways.^21^ Therefore, possibly, the virus binds to these cells without going through the lung and bronchus, where the ACE2 receptor is expressed much more than in external respiratory airway cells.^66^ Trapping the virus at the external respiratory cells could thus result in a lower infection probability, but a high virion concentration in the respiratory tract might increase the probability of spreading the virus.

## Supporting Information Available

Additional molecular dynamics and experimental FIDA and FCM results (PDF)
