# Supplementary material for: Two Receptor Binding Strategy of SARS-CoV-2 Is Mediated by Both the N-Terminal and Receptor-Binding Spike Domain
Source: J Phys Chem B. 2024 Jan 8;128(2):451–64. doi: 10.1021/acs.jpcb.3c06258 (PMC10801686; doi:10.1021/acs.jpcb.3c06258)
Supplement: Supplementary file 1 — jp3c06258_si_001.pdf [file jp3c06258_si_001.pdf]

SUPPORTING INFORMATION:

Two Receptor Binding Strategy of SARS-CoV-2 is Mediated by Both the N-Terminal and Receptor-Binding Spike Domain

Michele Monti*,[*,†,‡,∇] Edoardo Milanetti,[¶,‡,∇] Myrthe T. Frans,[§,∇] Mattia Miotto,[‡,∇] Lorenzo Di Rienzo,[‡,∇] Maksim V. Baranov,[§] Giorgio Gosti,[‖,‡] Arun Kumar Somavarapu,[⊥] Madhu Nagaraj,[⊥] Thaddeus W. Golbek,[#] Emiel Rossing,[@] Sam J. Moons,[@] Thomas J. Boltje,[@] Geert van den Bogaart,[§] Tobias Weidner,[#] Daniel E. Otzen,[⊥] Gian Gaetano Tartaglia,[†] Giancarlo Ruocco,[¶,‡] and Steven J. Roeters[*,#,△]

†*RNA System Biology Lab, Department of Neuroscience and Brain Technologies, Istituto Italiano di Tecnologia, Via Morego 30, 16163, Genoa, Italy*

‡*Center for Life Nanoscience, Istituto Italiano di Tecnologia, Viale Regina Elena 291, 00161, Rome, Italy*

¶*Department of Physics, Sapienza University, Piazzale Aldo Moro 5, 00185, Rome, Italy*

§*Molecular Immunology – Groningen Biomolecular Sciences and Biotechnology, Nijenborgh 7, 9747 AG, Groningen, The Netherlands*

‖*DHILab, Istituto di Scienze del Patrimonio Culturale, Sede di Roma, Consiglio Nazionale delle Ricerche, Via Salaria km 29300, Rome, 00010, Italy*

⊥*Interdisciplinary Nanoscience Center (iNANO), Aarhus University, Gustav Wieds Vej 14, 8000 Aarhus C, Denmark*

#*Department of Chemistry, Aarhus University, Langelandsgade 140, 8000, Aarhus C, Denmark*

@*Synthetic Organic Chemistry, Radboud University Nijmegen, Heyendaalseweg 135, 6525 AJ Nijmegen, The Netherlands*

△*Amsterdam UMC, Vrije Universiteit, Department of Anatomy and Neurosciences, De Boelelaan 1108, 1081 HZ, Amsterdam, The Netherlands*

∇*These authors contributed equally to the presented work.*

E-mail: michele.monti@iit.it; s.j.roeters@amsterdamumc.nl

Additional experimental data

1. Molecular dynamics simulations

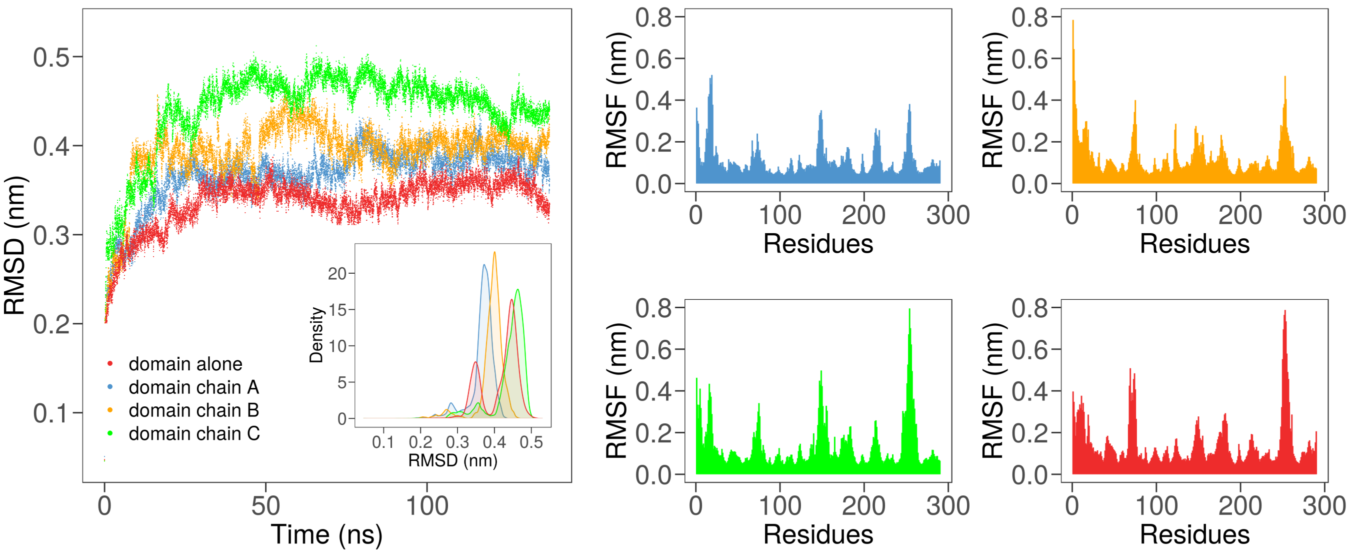

FIG. S1: **Comparison of the NTD dynamics in SARS-CoV-2 spike monomers and trimers.** Root mean square deviation (RMSD) and fluctuations (RMSF) as a function of time and chain residues, respectively, for the N-terminal domain (NTD) in a trimeric SARS-CoV-2 spike conformation, and as a isolated monomeric (chain A) NTD. "Chain A", "chain B" and "chain C" indicate the three NTDs during the molecular-dynamics simulation of the trimeric form of the spike protein, and, "domain alone" refers to the N-terminal domain considered alone in solution.

	fastDRH (kcal/mol)										Autodock (kcal/mol)
	PB1	PB3	PB4	GB1	GB2	GB5	GB6	GB7	GB8	mean	default parameters
p1	1.03	-9.23	-9.19	-9.91	-8.70	-8.45	-7.57	-7.84	-7.96	-8.61	-8.23
p2	-1.79	-7.45	-10.94	-17.00	-13.68	-13.59	-9.64	-11.61	-12.89	-12.10	-4.99

TABLE S1: p1: pose 1, which corresponds to the minimum energy configuration as calculated by Autodock; p2: pose 2, which corresponds to the minimum energy (calculated with Autodock) among the poses in which the region predicted by the Zernike method is involved in binding with SA. Note that the average value among the 9 parameter sets used by fastDRH was obtained by excluding the PB1 method, which yields results not in line with the other methods used.

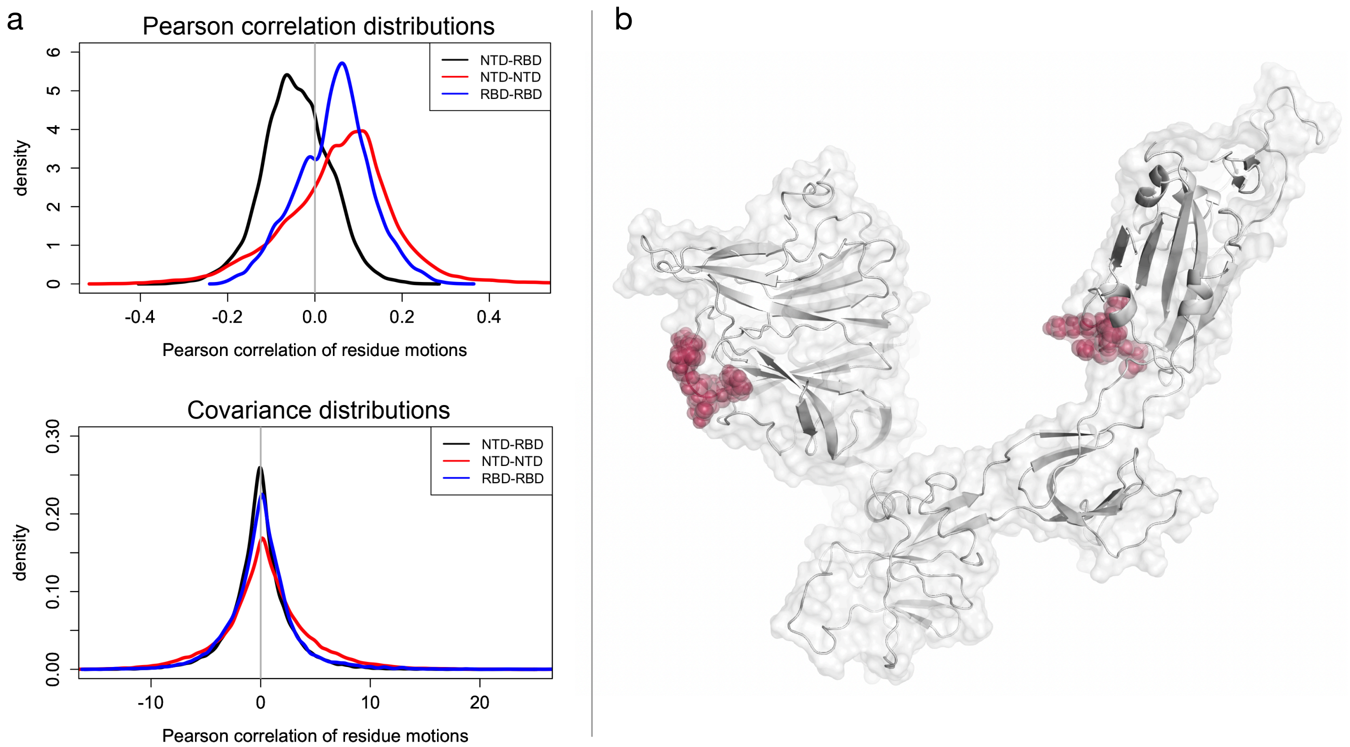

FIG. S2: **Analysis of the correlated motions between S1 residues.** **a** Density distribution of the Pearson correlation between the motion of all the couple of residues of the RBD, NTD and RBD-NTD. **b** Cartoon representation of SARS-CoV-2 spike protein S1 unit with residues having highest anti-correlated motion highlighted in red.

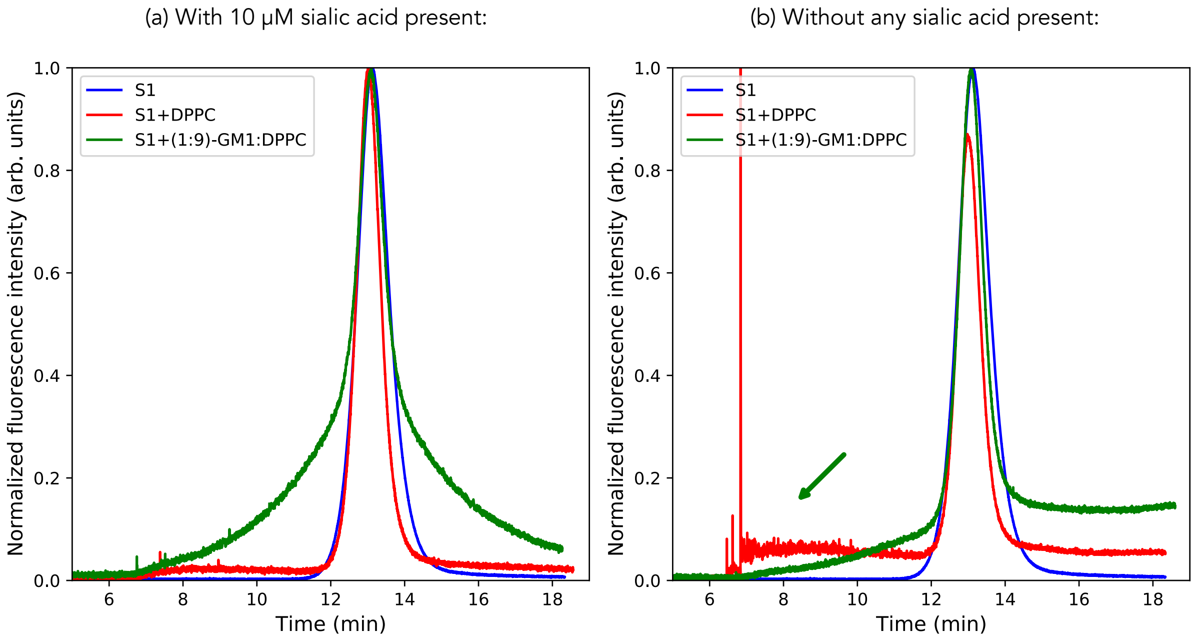

FIG. S3: **Effect of the addition of free sialic acid on the Taylograms recorded for the S1 segment of SARS-CoV-2.** **(a)** With 10 μM free sialic acid present, the Taylorgrams of the S1-DPPC sample indicates that there is no complex formation, while in figure **(b)**, in the absence of free sialic-acid molecules, nonspecific binding occurs (as indicated by spikes near the green arrow).

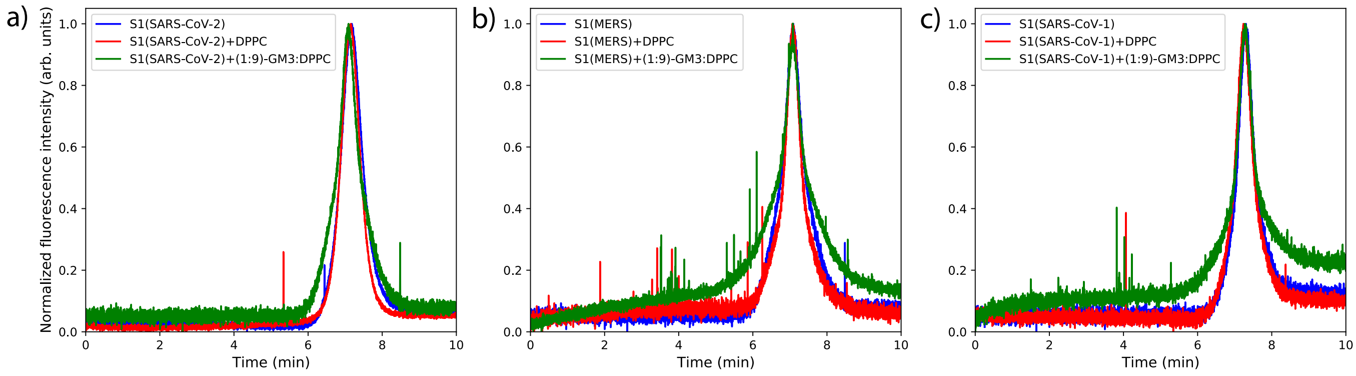

FIG. S4: **Taylorgram overlaps of pure 100 nM S1 protein, 10 µM sialic acid (to prevent non-specific interactions, see main text *Methods* section, and 200 µM (1:9)-GM3-DPPC or 200 µM DPPC vesicles, for three β-coronaviruses: a)** SARS-CoV-2, **b)** MERS, and **c)** SARS-CoV. Because Taylorgrams of different experiments cannot be overlapped well given differences in the exact circumstances in different FIDA experiments, the experiments are depicted as separate Taylorgrams depicted in main text Figure 3b) and in the current figure.

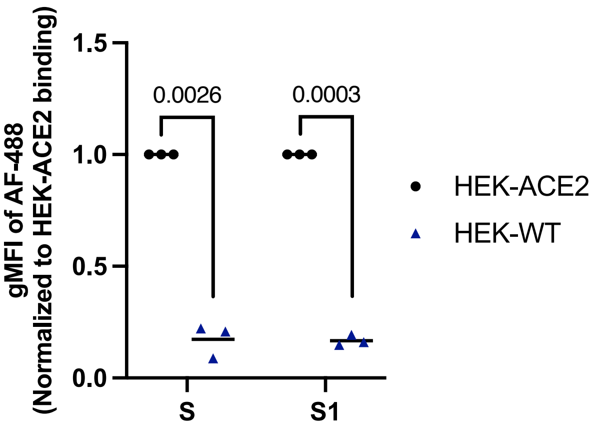

FIG. S5: **Comparison of S1-segment and full-length spike binding to HEK–wild-type (WT) vs. to HEK–ACE2 cells**. The remaining staining in wt cells is probably due to unspecific staining or a sub-optimal gating strategy. Similar to the experiment depicted in *Main Text* Figure 4d), the removal of one of the receptors involved in the two-receptor strategy of SARS-CoV-2 leads to a strong decrease in the spike (segment) binding efficiency. Two separate experiments showed that wild type (WT)-HEK cells express ~20% less SA than ACE2-HEK cells, even under the DMSO control conditions, so WT-HEK cells seem to have slightly less SA expressed at baseline (data not shown). The SA-expression difference is thus much to small to explain the strong S1 and S binding decrease observed here for WT- vs. ACE2-HEK cells.

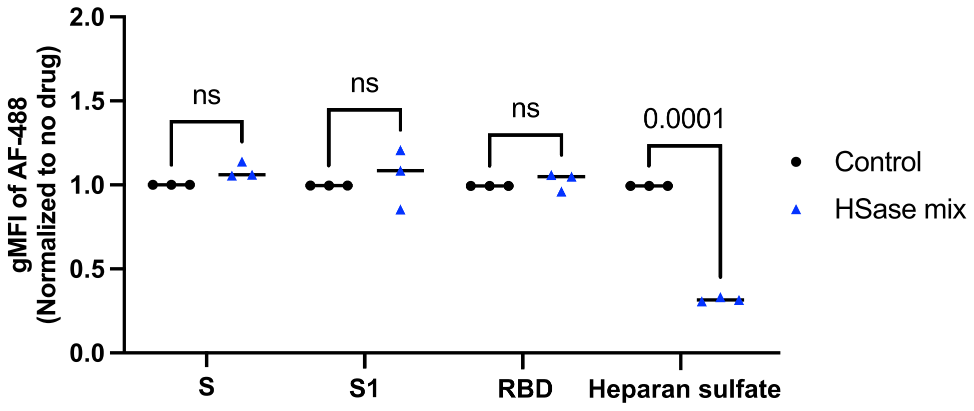

FIG. S6: **Flow cytometry data obtained for HSase-incubated HEK-ACE2 cells**. As opposed to what was previously found for other cell lines [1], for HEK-ACE2 cells, there is not a significant (i.e., a "ns") effect of the HSase incubation on the cell binding of any of the three spikes or spike segments.

[1] T. M. Clausen, D. R. Sandoval, C. B. Spliid, J. Pihl, H. R. Perrett, C. D. Painter, A. Narayanan, S. A. Majowicz, E. M. Kwong, R. N. McVicar, et al., Cell **183**, 1043 (2020).
